# Supplementary material for: Reliability of Xsens IMU-Based Lower Extremity Joint Angles during In-Field Running
Source: Sensors (Basel). 2024 Jan 29;24(3):871. doi: 10.3390/s24030871 (PMC10856827; doi:10.3390/s24030871)
Supplement: Supplementary file 1 [file sensors-24-00871-s001.zip › sensors-2821124-supplementary.pdf]

Supplementary Materials

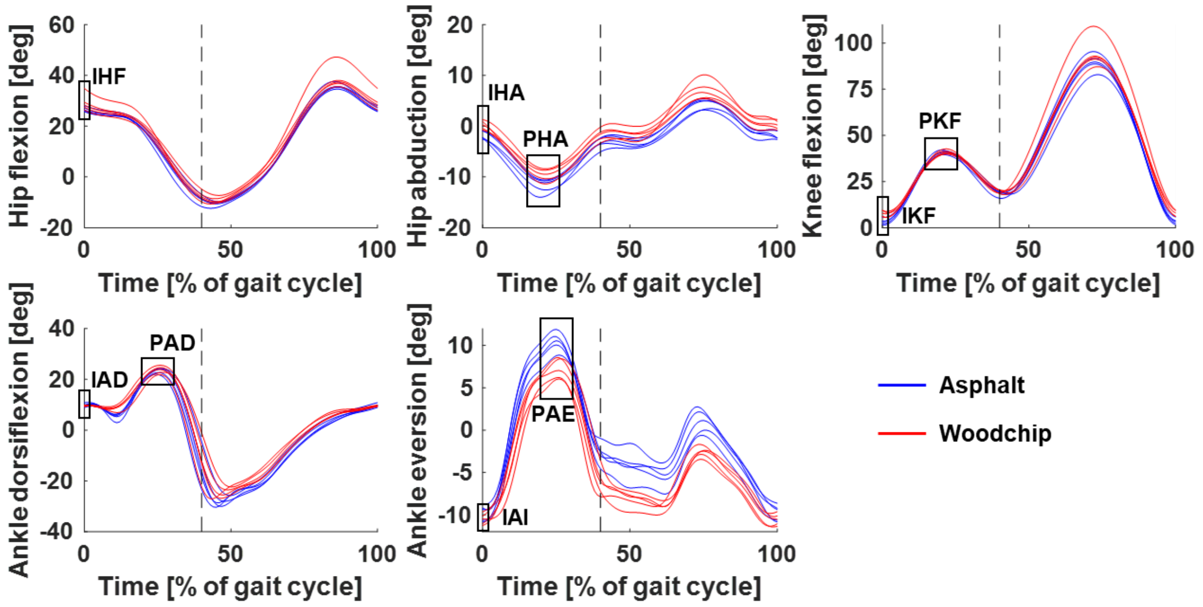

Figure S1. Xsens-based joint angles as a function of time during the running gait cycle for five testing days of one exemplary runner. Blue and red lines show the mean joint angle trajectories for this runner for each of the five testing days on either asphalt or the woodchip track, respectively. Time = 0% and 100% represent subsequent instances of the most anterior-posterior position of the right foot (approximate foot contact). The dashed vertical line represents the approximated toe-off at 40% of gait cycle duration. The rectangles illustrate the nine discrete joint angles that were investigated for their reliability: IHF = initial hip flexion, IHA = initial hip ab-/adduction, PHA = peak hip adduction, IKF = initial knee flexion, PKF = peak knee flexion, IAD = initial ankle dorsiflexion, PAD = peak ankle dorsiflexion, IAI = initial ankle inversion, PAE = peak ankle eversion.
